# Exploring the Association Between Urinary Incontinence and Depression Based on a Series of Large-Scale National Health Studies in Türkiye

**DOI:** 10.3390/jcm14155213

**Published:** 2025-07-23

**Authors:** Muhammed Furkan Dasdelen, Zehra Betul Dasdelen, Furkan Almas, Beyza Cokkececi, Pilar Laguna, Jean de la Rosette, Mehmet Kocak

**Affiliations:** 1 International School of Medicine, Istanbul Medipol University, Istanbul 34810, Türkiye; 2 Institute of AI for Health, Helmholtz Zentrum München, 85764 Munich, Germany; 3 Faculty of Engineering and Natural Sciences, Sabanci University, Istanbul 34956, Türkiye; 4 Department of Urology, Istanbul Medipol University, Istanbul 34810, Türkiye; 5 Institute of Urology and Clinical Oncology, Bashkir State Medical University, 450008 Ufa, Russia; 6 Department of Urology and Guangdong Key Laboratory of Urology, The First Affiliated Hospital of Guangzhou Medical University, Guangzhou 510120, China; 7 Department of Biostatistics and Medical Informatics, Istanbul Medipol University, Istanbul 34810, Türkiye; 8 Multi-Omics Design and Analysis Studio (MODAS-SABITA), Istanbul Medipol University, Istanbul 34810, Türkiye

**Keywords:** urinary incontinence, depression, health survey, longitudinal analysis, epidemiology, national, correlation, socio-economic, BMI

## Abstract

**Background**: Urinary incontinence (UI) and depression are prevalent conditions affecting millions globally and are significantly associated with various demographic, health, and socio-economic factors. This study examines the associations between UI and depression over a 14-year period using nationwide data. **Methods**: We analyzed cross-sectional data from the Turkish Health Studies Surveys conducted in seven different years between 2008 and 2022, including 125,276 participants aged 15 and older and excluding those with incomplete key health data. Variables included chronic conditions, BMI, depression severity (assessed by PHQ-8), socio-economic status, and lifestyle factors. Univariable and multivariable logistic regression models were used to investigate associations between UI and various risk factors over time. **Results**: The prevalence of UI and depression fluctuated over the 14 years, with a significant increase observed in 2014. Multivariate analysis confirmed a strong and consistent association between UI and depression across genders and age groups, even after adjusting for confounders. Higher depression severity increased the odds of experiencing UI. Age, multiple comorbidities, higher BMI, and lower socio-economic status were associated with an increased likelihood of UI. Obesity was a significant risk factor for UI in females but not in males. Urban living and higher education levels were inversely associated with UI. The simultaneous rise in UI and depression in 2014 may be linked to socio-economic changes during that period. **Conclusions**: The findings suggest a robust link between UI and depression, influenced by a complex interplay of health, demographic, and socio-economic factors, needing prospective studies to further investigate the causal pathway of these associations.

## 1. Introduction

Urinary incontinence (UI) is a common and often distressing condition that affects over 400 million people worldwide [1]. While it is often assumed to be a concern primarily for women and the elderly, UI can be seen across all age groups and sexes [2]. Studies indicate that up to 25–45% of women experience some degree of urinary incontinence, with the incidence rising with age [2,3]. In men, the prevalence is lower, estimated at around 2–11%, but also increases with age [2,4,5]. Cross-sectional and longitudinal studies have found associations between UI and certain risk factors, such as increased BMI [6,7,8], childbirth [8,9], menopause [8,10], and chronic conditions including diabetes, cardiorespiratory disorders, and musculoskeletal problems [11,12,13]. These increasing risk factors with age elevate the likelihood of developing UI in the elderly [8,12]. Regardless of age, UI remains bothersome for all, leading to a lack of self-confidence, embarrassment, disturbances in work or social life, and adverse psychosocial outcomes, including depression, social isolation, and anxiety [14,15].

Previous cross-sectional studies showing a strong association between UI, depression, and anxiety have been reported in European and Western countries [16,17,18,19,20,21]. Although the majority of these studies have been conducted on women, the association has also been demonstrated in men [20,21]. The causal direction of this relationship was better observed through longitudinal studies. Prospective studies found that both depression and anxiety are risk factors for UI [22,23,24,25,26,27,28,29], and UI can be a predictor of both [15,26,27,28], suggesting a potential bidirectional causality. Further research is needed to better understand this relationship, considering possible confounders such as demographics, environmental factors, and comorbidities in different populations.

In a previous study, we examined the relationship between depression, anxiety, and urinary incontinence in the presence of various comorbidities [30]. We found that the prevalence of urinary incontinence increases as the level of psychological discomfort rises in the Turkish population. This relationship might be a direct or indirect cause-and-effect relationship, which can be better observed through longitudinal studies.

In the present study, we examined data from the Türkiye Health Interview Survey, collected in seven different years between 2008 and 2022. It includes a total of 125,276 participants representing the whole population at the time of collection. Our first aim is to discover the trend in urinary incontinence and depression prevalence over this 14 year period and explore external factors influencing this relationship. Our second aim is to investigate the consistency of the association between depression and urinary incontinence in each cross-sectional dataset, considering the comorbidities and characteristics of participants.

## 2. Materials and Methods

### 2.1. Data Collection

As adopted modules from the Eurostat European Health Interview Survey (EHIS) and conducted as a part of EHIS waves, the Türkiye Health Survey was carried out first in 2008 and was implemented every two years until 2016, and at three-year intervals afterwards in 2019 and 2022.

The data collection technique has been detailed in earlier studies [30,31,32]. In summary, a stratified two-stage cluster sampling approach was employed. The sampling spanned all geographical regions and included every resident in Türkiye. The total sample size has varied, from 7910 in 2008 to 11,179 households in 2022. Interviews were conducted face-to-face, with the first survey taking place in April 2008. Subsequent surveys occurred over one month in May–June during 2010 and 2012, over three months in August–October during 2014 and 2016, and in September–December in 2019 and 2022. The participants provided informed consent before joining the study. Ethical approval was granted by the Istanbul Medipol University Ethics Committee (application number 10840098-604.01.01-E.53819). All procedures were carried out in compliance with the Declaration of Helsinki.

The survey’s objective is to periodically collect data on the health indicators of Türkiye, enabling the monitoring of changes in the population’s health status and its determinants. The survey included three primary modules: health status, health care services, and health determinants. Here, we primarily used health status sections which encompass diseases, chronic conditions, physical and sensory impairments, pain, and mental health issues.

### 2.2. Survey Weights and Calibration

All analyses were performed with individual survey weights supplied by TURKSTAT. These weights were constructed in three steps that follow the European Health Interview Survey (EHIS) methodology guide: (i) the inversion of each person’s selection probability in a stratified two-stage design (block → household → individual); (ii) a non-response adjustment within rural/urban × NUTS strata; and (iii) a first-stage calibration that forces the weighted sample to reproduce the official mid-year population totals for sex and five-year age groups published by the Address-Based Population Registration System (ABPRS). Because fieldwork for some waves extended into the fourth quarter and urban–rural information was not available in some years, we performed additional post-survey raking to the final ABPRS counts of the corresponding calendar year. Using the survey package in R, a design object was specified with household ID as the primary sampling unit, Nomenclature of Territorial Units for Statistics-1 (NUTS-1) as the stratum, and the TURKSTAT weight as the starting weight. The calibrated weights were subsequently applied in every prevalence estimate and logistic regression model.

### 2.3. Variables and Categorization

Participants self-reported their chronic conditions, responding with ‘Yes’, ‘No’, ‘Do not know’, or ‘Refuse to answer’. We included the following chronic conditions in our analysis: asthma, COPD, myocardial infarction (MI), chronic heart failure, hypertension, coronary artery disease, stroke, osteoarthritis, diabetes mellitus, cirrhosis, and depression. The options ‘Do not know’ and ‘Refuse to answer’ were omitted after 2012. Participants were also given the option not to answer a specific question. Those who answered ‘Yes’ were considered to have the disease. Individuals with any of the following cardiovascular conditions were categorized as having a cardiac disease: myocardial infarction, hypertension, chronic heart failure, or coronary artery disease. The PHQ-8 scale was utilized to assess the scale of depressive symptoms. The distinction between urban and rural lifestyles was only included for the years 2008, 2010, and 2012. Regional categorization was based on the Nomenclature of Territorial Units for Statistics (NUTS), which divides major socio-economic regions.

Education levels were classified according to the International Standard Classification of Education (ISCED) 2011 and Eurostat’s guidelines [33]. ISCED 2011 levels 0–2 were classified as low education, levels 3–4 as medium education, and levels 5–8 as high education. For body mass index (BMI), categories were defined as follows: underweight for a BMI less than 18.5, normal weight for a BMI ranging from 18.5 to 24.9, overweight for a BMI from 25.0 to 29.9, and obese for a BMI of 30.0 and above.

### 2.4. Eligibility Criteria

**Figure 1:**
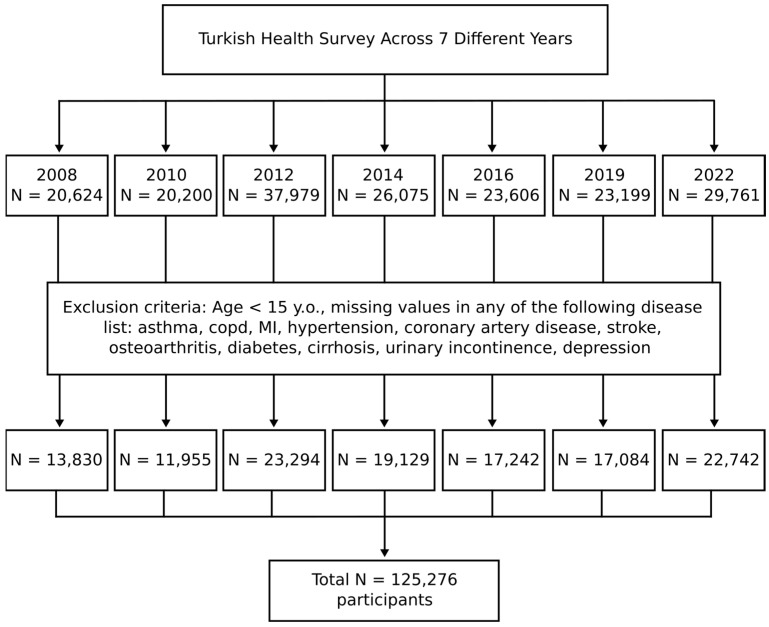
Flow chart of the study. A total of 125,276 participants were included in the study after applying the exclusion criteria to the participants from 7 different years. For each year, separate multivariable logistic regression models were utilized to evaluate the associations between urinary incontinence and the independent variables.

Initially, individuals under 15 years of age were excluded (Figure 1). Additionally, we excluded cases with missing values for chronic diseases.

### 2.5. Statistical Analysis

We utilized univariable and multivariable logistic regression models to examine the relationship between urinary incontinence (UI) and various factors. For biological and demographic factors, we employed separate multivariable logistic regression models for each year, with UI as a binary dependent variable and other chronic conditions, age, and BMI as independent variables. For lifestyle–socioeconomic factors and depression severity, we initially applied univariable logistic regression and subsequently adjusted for age and chronic conditions using multivariable logistic regression. We used the Chi-squared test to analyze prevalence differences according to income levels.

For UI and depression prevalence trends over years, we fitted two different interrupted time series regression models. We assumed an event interrupting the natural trend of the UI or depression prevalence between 2012 and 2014. We wrote the prevalence as a function of time as follows:

$$Prevalence\left(t\right)=\beta_{0}+\beta_{1}\times t+\beta_{2}*event+\beta_{3}\times t_{after\;event}+\varepsilon (t)$$

where $\beta_{0}$ is the baseline level of the prevalence and $\beta_{1}$ is the trend of prevalence before the event. $\beta_{2}$ is the level change immediately after the event while $\beta_{3}$ is the change in trend of prevalence after the event. Event is a binary variable indicating whether time t is before (0) or after (1) the event. ε(t) stands for the error term or residuals. Prais–Winsten estimation from the Prais package in R was utilized to take care of autocorrelation.

For a detailed analysis of the relationship between BMI and UI, we performed a restricted cubic spline (RCS) analysis separately for each gender to explore potential non-linear associations. Data from all years were combined, and individuals with missing BMI values or extreme values (BMI < 15 or BMI > 45) were excluded (excluded individuals: *n* = 1310 for males; *n* = 4163 for females). The final analysis included 55,051 males and 64,755 females. Knots were placed at the quantiles of the BMI distribution within each gender subgroup to adequately capture BMI variability. The transformed BMI variables were included in a multivariable logistic regression model with UI as the dependent variable, adjusting for age and chronic conditions (asthma, COPD, cardiac disease, stroke, osteoarthritis, diabetes mellitus, cirrhosis, and depression). For model predictions, age was set to its mean value and all binary comorbidity variables were set to 0. A post-hoc ANOVA was conducted to assess the significance of non-linearity. In calculating odds ratios, a BMI of 25 was used as the reference point.

A *p* value of less than 0.05 was considered statistically significant throughout the study. Bonferroni correction was applied for multiple comparisons and indicated where applicable. Statistical analysis and visualizations were conducted using Python v3.10. Prevalence estimates and logistic regression analysis were conducted in R v4.5. For pooling odds ratios from different years, we used random effects models provided by the *metafor* package in R.

Codes used for analysis are shared in https://github.com/mfdasdelen/Turkish_Health_Survey_analysis to ensure reproducibility.

## 3. Results

After applying the exclusion criteria across data from seven different years, a total of 125,276 participants were included in the study. The highest number of participants was recorded in 2012, while the lowest was in 2010 (Figure 1).

The demographic and medical characteristics of the participants in each year are demonstrated in Table 1. Notably, gender distribution showed a consistent pattern, with females comprising over half of the participants each year except 2008. Moreover, age distribution revealed a significant decrease in the youngest age group (15–24 years) over time, from 23.92% of the total participants in 2008 to 19.46% in 2022, while the proportion of participants aged higher than 55 increased from 18.38% in 2008 to 25.45% in 2022.

In terms of education, the proportion of individuals in the low ISCED category consistently decreased, along with increases in the medium and high categories, indicating an overall upward trend in the education level of the population. Marital status distribution demonstrated a steadily decreasing trend in the married population.

**Figure 2:**
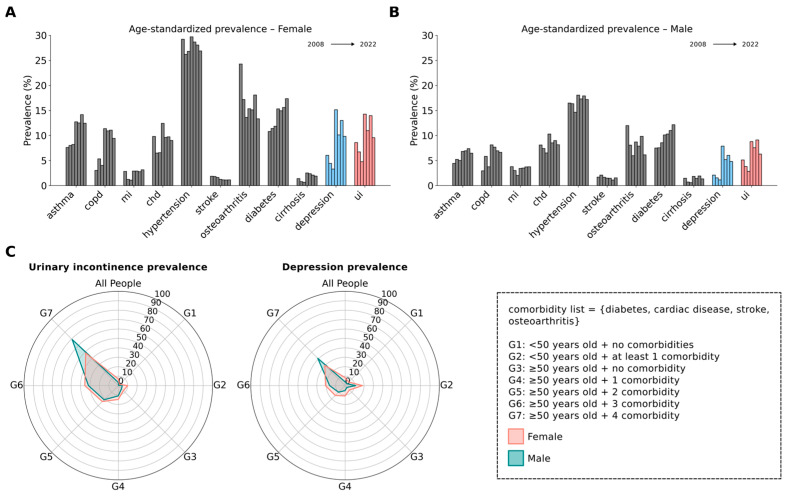
Urinary incontinence and depression prevalence increased with age and number of comorbidities in both sexes. Age-standardized prevalence of UI and depression followed similar trends over the years for both sexes (**A**,**B**). Each bar represents the prevalence of the condition in the following year order: 2008, 2010, 2012, 2014, 2016, 2019, and 2022. Chronic diseases showed different temporal trends across years. The prevalence of UI and depression varied across different age and comorbidity subgroups (**C**). Separate radial plots were generated for UI and depression, each illustrating seven subgroups defined by age and number of comorbidities. Within the radial plots, each circle represents a 10 percent interval.

Population-level prevalence estimates of chronic diseases for each year are presented in Table 1. However, because population age structure changes over time, we additionally calculated age-standardized prevalence rates to allow for more accurate trend comparisons (Figure 2A,B, Supplementary Table S1). The age-standardized prevalence of urinary incontinence (UI) demonstrated notable variation in both sexes across years. Among females, prevalence peaked at 14.25% in 2014, rising from 8.57% in 2008, before declining slightly to 10.96% in 2016. A marked increase was also observed between 2016 and 2019. Similarly, depression rates showed substantial fluctuation, with a pronounced peak of 15.13% in 2014 followed by a decrease to 9.85% in 2022. In males, the prevalence of UI exhibited a comparable peak at 8.78% in 2014, up from 5.10% in 2008, and declined to 6.28% by 2022. Depression prevalence in males also peaked at 7.86% in 2014, mirroring the trend seen in females. Throughout the study period, females consistently reported higher rates of both UI and depression compared to males across the majority of years. Notably, UI and depression followed closely aligned temporal patterns when compared to other comorbidities plotted on the same graph.

The prevalence of UI and depression was further analyzed in subgroups with varying comorbidities (Figure 2C). Both UI and depression increased in parallel with age and the number of comorbidities. Participants aged 50 years and with multiple comorbidities, including diabetes, cardiac disease, stroke, and osteoarthritis, exhibited the highest prevalence rates of both UI and depression. Having a comorbidity, even at below 50 years of age, dramatically increased the prevalence of depression and UI.

**Figure 3:**
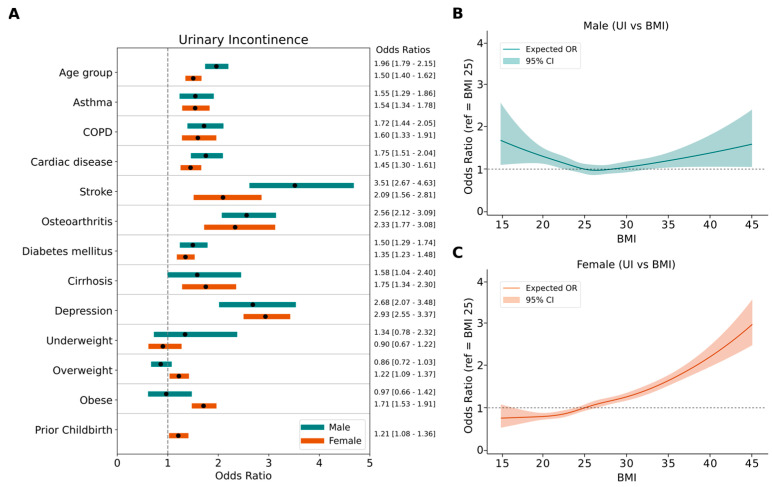
Age and chronic diseases associated with increases in urinary incontinence. Multivariable logistic regression was conducted separately for each year, with urinary incontinence as the dependent variable. Odds ratios were pooled for each independent variable. Age and all chronic conditions significantly increased the odds of urinary incontinence in both genders, while obesity was only associated with urinary incontinence in females (**A**). Restricted cubic spline (RCS) curves were drawn to visualize the UI-BMI association in males (**B**) and females (**C**). Solid lines represent expected odds ratio while shades show 95% confidence intervals.

We next analyzed the associations between various factors and UI using logistic regression models. First, we evaluated age, BMI, and chronic conditions, which are all biological components. Separate multivariable logistic regression models were applied for each year and the odds ratios for each independent variable were then pooled, as represented in Figure 3. Detailed odds ratios for individual years were included in Supplementary Figure S1.

Multivariable logistic regression analysis revealed a strong and consistent relationship between UI and depression across multiple years for both genders (Figure 3, Supplementary Figure S1). The pooled odds ratio of depression was 2.93 [2.55–3.37, 95% CI] in females and 2.68 [2.07–3.48 95% CI] in males, indicating a significant association. Furthermore, people with higher depression severity had higher odds ratios for UI (Table 2). Aging remained a primary contributor to UI, with pooled odds ratios of 1.50 [1.40–1.62, 95% CI] for females and 1.96 [1.79–2.15 95% CI] for males. Additionally, several other chronic conditions, including cardiac diseases, stroke, diabetes mellitus, osteoarthritis, COPD, asthma, and cirrhosis revealed significant associations with UI in both genders. Prior childbirth increases the prevalence of UI in females (OR 1.21 [1.08–1.36, 95% CI]). Interestingly, obesity was significantly associated with UI in females but not in males.

To thoroughly evaluate the relationship between urinary incontinence (UI) and BMI, we pooled participants from all years and conducted a restricted cubic spline (RCS) regression (Figure 3B,C). The model revealed an almost linear increasing trend for females (*p* value for non-linearity = 0.48), while a U-shaped graph was observed for males (*p* value for non-linearity < 0.01).

For lifestyle and social factors, we applied both univariable and multivariable logistic regression, adjusting for age, BMI, and other comorbidities. The adjusted odds ratio for an urban lifestyle was 0.76 [0.66–0.88] for females and 0.59 [0.47–0.74] for males (Table 2). People with medium and high education levels had lower odds of having UI compared to those with low education in both genders. Marital status was not significant in males, while married females exhibited higher adjusted odds ratios (OR 1.13 [1.05–1.22, 95% CI]). However, the observed association between marital status and UI in females was attenuated and no longer statistically significant after adjustment for previous pregnancy in the multivariable model (Supplementary Table S2). The use of depression or anxiety medication was not significantly associated with UI in either sex after adjusting for comorbidities.

**Figure 4:**
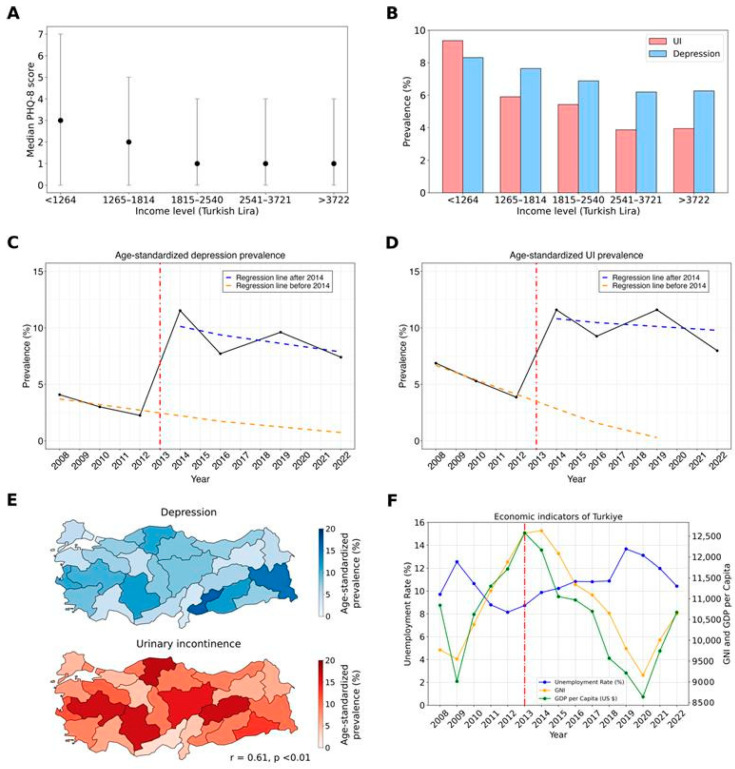
Effect of income and macroeconomic indicators on UI and depression prevalence. Median PHQ-8 scores by income level (**A**). Dots represent median values, while the gray line indicates the lower and upper quartiles. Prevalence of depression and urinary incontinence (UI) by income level, showing decreases in both conditions as income increases (**B**). Interrupted time series analyses for depression (**C**) and UI (**D**). The vertical red line marks the event that disrupts the natural trend of the diseases. The orange dashed line depicts the underlying trend of prevalence prior to and projected for after the event, assuming no disruption had occurred. The blue dashed line shows the trend in prevalence following the event. Changes in the levels between the orange and blue lines indicate the immediate impact of events, while changes in the slopes represent their ongoing effects. Distribution of depression and UI prevalence across NUTS-2 regions (**E**). Visualization of the unemployment rate, GNI, and GDP per capita (**F**). Data sourced from TurkStat.

We observed that as individuals’ income levels increased, median PHQ-8 scores decreased (Figure 4A), with low-income individuals exhibiting higher PHQ-8 scores. Moreover, people with low incomes experienced higher rates of depression and UI, and the prevalence of both conditions decreased as income levels rose (Figure 4B). Significant differences in UI prevalence between income levels are documented in Supplementary Table S3. Additionally, the distribution of depression and UI prevalence across statistical regions (NUTS2) shows a high correlation (Figure 4E, r = 0.61, *p* < 0.01).

In addition to the strong association between UI and depression, we observed that the prevalence of both conditions followed the same trend over the years in both genders (r = 0.96, *p* < 0.01) (Figure 2A,B and Figure 4C,D). Although the prevalence of UI and depression consistently decreased between 2008 and 2012, we noted a sudden surge in 2014 followed by some fluctuations. For this reason, we applied segmented regression to the interrupted time series data. We assumed that a change occurred between 2012 and 2014 and tested its impact on the prevalence of depression and UI. The model showed good fit for both UI (residual standard error = 0.47, adjusted R^2^ = 0.99) and depression (residual standard error = 0.82, adjusted R^2^ = 0.98). Prior to 2014, there was a downward trend in the prevalence of depression and UI, indicated by the orange dashed lines. In 2014, there was a sharp increase followed by fluctuations towards a decreasing trend, although levels remained higher than before the event. Generally, the time factor correlates negatively in both the depression and UI models (Supplementary Table S5). Before 2014, UI showed a 1.3 unit drop per year (*p* = 0.03), while depression showed a 0.5 unit decline (*p* = 0.42). After 2014, the time factor correlates negatively with depression but contributes positively to the prevalence of UI. This is also visualized by the blue lines, where depression exhibits a lower negative slope.

To understand the sudden increase in the prevalence of depression and UI in 2014, we thoroughly examined nationwide events and socio-economic changes in Turkey (Figure 4F). According to data from TurkStat, Turkey’s unemployment rate began to rise in 2013, reaching 8.73%, and peaked in 2019 at 13.67%. Similarly, Turkey’s GDP per capita and GNI began to decline after 2013, indicating an economic downturn. There was a positive correlation between unemployment rate and the prevalence of UI and depression; however, it was not statistically significant (r = 0.64, *p* = 0.12 for UI; r = 0.50, *p* = 0.25 for depression).

## 4. Discussion

In the present study, we extensively analyzed the trends and associations between urinary incontinence (UI) and depression in a large, representative national sample from the Türkiye Health Interview Survey spanning from 2008 to 2022. Our findings underscore a significant and consistent association between UI and depression across different years, genders, and age groups, highlighting a robust link regardless of the underlying demographic and health status factors. Our analysis revealed that higher depression severity correlates with an increased likelihood of UI, reinforcing the potential bidirectional nature of this relationship. The age and number of comorbid conditions also emerged as critical factors, with older individuals and those with multiple chronic conditions showing higher prevalences of both UI and depression. Notably, we observed that both conditions displayed parallel trends over the study period, with a marked increase in prevalence in 2014, followed by fluctuations but remaining at elevated levels compared to the initial years of the survey. This surge aligns temporally with significant socioeconomic changes in Turkey, suggesting that broader societal stress may influence these health outcomes.

Both cross-sectional and longitudinal studies in the current literature have consistently demonstrated a strong bidirectional association between UI and psychological discomfort, particularly depression, across both genders [15,16,17,18,19,20,21,22,23,24,25,26,27,28,29,30]. Individuals with UI often experience increased levels of psychological distress, compounded by the physical limitations and social stigmatization associated with UI, while depression can further exacerbate the perception of UI severity and hinder the effective management of both UI and its comorbidities [16,34]. In line with these findings, our previous study revealed a similar pattern, where depression prevalence increased alongside UI, particularly in middle-aged women [30]. In the present research, we provide further evidence supporting this firm relationship between UI and depression by using a large, nationally representative dataset spanning multiple years. Notably, our results not only reaffirm this well-established association but also expand on previous research by incorporating the impact of additional contributing factors such as aging, chronic conditions, and lifestyle elements. Moreover, this comprehensive analysis was conducted in light of macroeconomic and temporal contexts, which may reflect broader societal or economic changes influencing the trends during that 14 year period. Thus, it enhances our understanding of how these two conditions evolve together over time and across different demographic and medical subgroups.

Previous studies have attributed the association between UI and chronic conditions such as diabetes and cardiovascular diseases to their role in compromising pelvic floor function [2,35]. Neurodegenerative diseases like Parkinson’s and Alzheimer’s or stroke have also been blamed for contributing to UI through both disrupted neurological pathways and overall decline in motor control [35]. Functional impairments and mobility problems are associated with UI via decrease in muscle quality [13]. By conducting multivariable analysis, we have shown that different chronic conditions are associated with UI and the increased likelihood of having it (Figure 3). It is also observed and proven that the presence of chronic conditions, particularly when multiple comorbidities are involved, has been linked to greater psychological distress, which subsequently leads to depression [29]. In parallel with the existing literature, chronic illnesses significantly impacted both urinary incontinence (UI) and depression prevalence in our study population (Figure 2).

Interestingly, as shown in Figure 2, the current findings reveal that both sexes follow a similar trajectory, with no significant gender differences in the relationship between comorbidities, UI, and depression. However, a distinctive feature of this study is the critical role of age in modulating these associations. Before the age of 50, comorbidities exert a relatively minimal impact on the development of UI and depression. In contrast, after 50, the presence of multiple chronic conditions markedly elevates the risk of both UI and depression, underscoring the compounded effect of aging and comorbidities.

We observed that aging, cardiac disease, stroke, and COPD had higher odds ratios for UI in males compared to females, while obesity was a significant risk factor only for females (Figure 3A). Thus, we further evaluated the gender-specific relationship between UI and BMI. Previous studies have shown that obesity is a modifiable risk factor for UI. However, only a limited number of studies have focused on gender-specific analysis of the relationship between BMI and UI. In line with a previous nationwide longitudinal study [36], we found that BMI is linearly correlated with UI in females but not in males (Figure 3B,C). The mechanism by which obesity increases the risk of UI is explained by multiple factors [36,37]. Excess body weight leads to increased abdominal pressure, which in turn augments bladder pressure and shifts urethral position, directly contributing to stress urinary incontinence (SUI) and exacerbating symptoms of detrusor instability and overactive bladder. Furthermore, obesity induces chronic strain and mechanical stress, stretching and weakening pelvic floor muscles, nerves, and other supporting structures, thus impairing pelvic organ function. This biomechanical stress is compounded by biochemical changes. Obesity is associated with systemic inflammation and oxidative stress, which promote vascular damage and sclerosis of the pelvic floor muscles and bladder tissues. These physiological changes are particularly evident in women, given their unique pelvic anatomy and factors such as pregnancy, childbirth, and menopause, which further damage pelvic tissues and diminish muscle tone and function. Increased chances of having UI in underweight men can be explained by an unmeasured covariate. Chen et al. [36] suggested that this covariate could be frailty, which increases disability and hospitalization and consequently UI rates. However, it should be acknowledged that frailty caused by underweight should have affected both genders equally. Further analysis with UI subtypes and frequency should be conducted to understand gender differences in the BMI–UI relationship since the dominant type of UI is different for males and females. A recent women-only cohort study showed that depression is associated with subsequent mixed UI and urgency, while only stress UI is prospectively associated with depression [38].

Economic factors have been recognized as significant determinants of mental health for a long time. Thoits and Hannan et al. [39], one of the first research groups, established that depression tends to inversely correlate with income, a finding supported by later empirical studies demonstrating that individuals with lower personal or household income are at an increased risk of depression [40,41,42]. Conversely, a recent Chinese large-scale survey study revealed a U-shaped correlation where mental health issues are particularly pronounced at both low and high ends of the socioeconomic spectrum [43]. In contrast, our findings, described in Figure 4A, showed that PHQ-8 scores decreased with increasing income up to a certain threshold, after which they plateaued.

Similarly, the prevalence of UI is influenced by economic conditions. Research shows that lower income and socioeconomic status contribute to higher rates of UI, partly due to limited access to healthcare resources and higher levels of psychological stress [44,45].

Not only economic but also socioeconomic factors have been shown to impact the prevalence of both UI and depression. Socioeconomic factors affecting UI consist of education, occupation, income, economic hardship and housing tenure, and poverty–income ratio [43,44], and the ones affecting depression are income, marital status, economic hardship (EHQ), financial threat (FTS), and financial well-being (FWBS) [41,43,46].

Our analysis, as depicted in Figure 4, highlights a notable shift in the prevalence of UI and depression in Turkey between 2012 and 2014. During this period, Turkey experienced economic difficulties and social unrest. Recent studies have highlighted that socio-political events and social unrest contribute to major mental health burden, especially depression and post-traumatic stress [47,48]. These findings underscore that the observed changes in UI and depression prevalence are not solely attributable to fluctuations in GDP per capita but are likely influenced by a complex interplay of economic and sociopolitical factors. The current literature comprises several studies supporting the correlation at the individual level while indicating the intricate nature of this relationship at the national level [49,50]. While our analysis revealed a link between microeconomic hardship and the prevalence of UI and depression, we were unable to establish a corresponding association at the macroeconomic level.

We discovered that a history of childbirth can increase the prevalence of UI among women, regardless of whether they have had a cesarean section (C-section) or a vaginal delivery. However, it was observed that vaginal delivery increases UI prevalence more than C-sections [51]. Given that Turkey’s fertility rate is declining each year [52] and C-section rates are as high as 60% [53]—with an increasing trend—it can be expected that UI prevalence may decrease. Apart from the surge in 2014, our segmented model (Figure 4D) suggests a decrease in UI prevalence, particularly among the female population. This difference in prevalence between genders may be explained by shifts in both fertility rates and childbirth practices.

An incidental finding in our dataset is the worrisome increase in the prevalence of diabetes mellitus (DM) in Turkey. DM is one of the major causes of mortality and morbidity and its prevalence is expected to rise globally [54]. In our dataset, there is a linear increase in DM prevalence, rising from 5.69% to 11.20% over 14 years. Our findings support a study that analyzed all electronic health records in 2020, which reported a DM prevalence of 11.12% based on lab-based diagnostic criteria [55]. Nationwide, appropriate, preventive screening and treatment strategies should be implemented.

Our results should be interpreted in the context of a few potential limitations. First, being an observational study, it is inherently limited to identifying associations and correlations rather than establishing causality. This restricts our ability to definitively state that one factor causes another, as relationships may be influenced by unknown or unmeasured variables. Second, as suggested, the use of nationally representative data may reflect the influence of country-specific economic and sociopolitical contexts, limiting the generalizability of our findings. Third, the health survey data does not contain measures to characterize UI or the quantity of urine lost per episode of incontinence. These are important dimensions for understanding the mechanism of UI and its risk factors in both genders. The presence of chronic conditions are all patient-reported outcomes (PROs) and irrespective of doctor’s confirmation or formal diagnosis. Additionally, while we observed a notable surge in the prevalence of depression and UI following an interruption in 2014, the observational nature of the study constrains our ability to pinpoint and fully explain the causative external factors or how they exert their influence on UI prevalence. The inability to clarify the mechanisms through which these external factors impact UI and depression underscores the need for further research. It remains unclear whether any changes occurred in survey methodology between 2012 and 2014; however, as the survey was administered by a governmental organization with standardized protocols, we do not anticipate any significant deviations in data collection procedures.

Despite the limitations, the present study’s strengths are evident through the comprehensive and longitudinal analysis of data collected over seven distinct years, involving a large and diverse sample size that reflects the general population of Türkiye. Such a longitudinal approach allowed us to not only confirm the high correlation between UI and depression but also to explore the interaction with various other chronic conditions and demographic factors. The extensive list of variables considered in our analysis—ranging from biological factors like age, body mass index (BMI), and a spectrum of chronic conditions, to socioeconomic factors including education levels, marital status, and urban vs. rural living—enhanced our understanding of the complex interplay between these elements and their impact on UI. This broad variable spectrum strengthens the study’s ability to provide a holistic view of the health landscape over an extended period, making it a valuable resource for public health assessments and policymaking.

## 5. Conclusions

Conducting nationwide health surveys at regular time intervals is an effective strategy to capture much needed data of disease prevalence in a spatiotemporal manner. They guide us in describing the current state of diseases and in assessing the impact of our health policies and interventions on various levels. The present study is a good example of such utilization of national survey data, showing the need for a more holistic approach towards the diagnosis, treatment, and follow-up of urinary incontinence, as our study suggests a complex association between UI and depression and patient characteristics, which should be taken into account in disease prevention and management.

## Figures and Tables

**Table 1:** Characteristics of participants by year. Counts are unweighted (raw numbers), while percentages are weighted using individual survey weights to reflect nationally representative prevalence estimates based on the complex survey design.

Variable	2008 *n* = 13,830	2010 *n* = 11,955	2012 *n* = 23,294	2014 *n* = 19,129	2016 *n* = 17,242	2019 *n* = 17,084	2022 *n* = 22,742
Gender							
Female	7475 (49.90%)	6977 (50.50%)	13,411 (51.15%)	10,408 (50.29%)	9574 (50.27%)	9300 (50.26%)	11,771 (50.31%)
Male	6355 (50.10%)	4978 (49.50%)	9883 (48.85%)	8721 (49.71%)	7668 (49.73%)	7784 (49.74%)	10,971 (49.69%)
Age							
15–24	2757 (23.92%)	2378 (23.00%)	4625 (22.29%)	3388 (21.73%)	2905 (21.33%)	2730 (20.26%)	3825 (19.46%)
25–34	3158 (23.62%)	2424 (23.38%)	4541 (22.70%)	3661 (21.74%)	3006 (20.62%)	3070 (19.91%)	4077 (19.34%)
35–44	2741 (19.22%)	2298 (18.58%)	4447 (19.09%)	3768 (19.43%)	3444 (19.92%)	3395 (19.63%)	4628 (19.49%)
45–54	2271 (14.87%)	2080 (15.44%)	3981 (15.36%)	3332 (15.49%)	3007 (15.61%)	2918 (15.97%)	3901 (16.26%)
55–64	1501 (9.46%)	1411 (10.36%)	2870 (10.80%)	2555 (11.09%)	2368 (11.59%)	2513 (12.42%)	3167 (12.75%)
65–74	876 (5.55%)	855 (5.68%)	1778 (5.99%)	1498 (6.41%)	1545 (6.72%)	1590 (7.41%)	2183 (8.19%)
75+	526 (3.37%)	509 (3.57%)	1052 (3.77%)	927 (4.11%)	967 (4.20%)	868 (4.40%)	961 (4.51%)
Asthma	692 (4.57%)	659 (5.16%)	1270 (4.98%)	1628 (7.81%)	1499 (7.73%)	1665 (8.81%)	1872 (7.97%)
COPD	299 (2.06%)	525 (4.32%)	702 (2.87%)	1616 (7.64%)	1369 (7.19%)	1373 (7.01%)	1508 (6.41%)
Myocardial infarction	260 (1.78%)	150 (1.21%)	240 (0.94%)	421 (2.01%)	422 (2.03%)	434 (2.17%)	561 (2.36%)
Coronary heart disease	889 (5.98%)	599 (4.45%)	1113 (4.25%)	1729 (8.39%)	1294 (6.47%)	1338 (7.06%)	1494 (6.31%)
Hypertension	2196 (14.18%)	1843 (13.09%)	3646 (13.06%)	3535 (15.86%)	3269 (15.61%)	3167 (16.05%)	3831 (15.80%)
Any cardiac disease	2753 (18.11%)	2243 (16.32%)	4325 (15.83%)	4407 (20.34%)	3865 (18.83%)	3867 (19.87%)	4589 (19.04%)
Stroke	165 (1.04%)	155 (1.14%)	247 (0.95%)	165 (0.84%)	167 (0.86%)	141 (0.78%)	219 (0.94%)
Osteoarthritis	1782 (11.84%)	1160 (8.29%)	1706 (6.26%)	1773 (7.91%)	1608 (7.59%)	2147 (10.96%)	1889 (7.88%)
Diabetes	897 (5.69%)	857 (6.24%)	1834 (6.72%)	1996 (8.88%)	1878 (8.93%)	1961 (10.02%)	2743 (11.20%)
Cirrhosis	150 (1.04%)	64 (0.50%)	99 (0.41%)	340 (1.59%)	292 (1.44%)	294 (1.57%)	301 (1.28%)
Depression	582 (3.83%)	403 (2.86%)	540 (2.02%)	2242 (10.88%)	1412 (7.13%)	1701 (8.88%)	1637 (6.83%)
Urinary incontinence	620 (4.25%)	445 (3.16%)	612 (2.31%)	1619 (7.41%)	1219 (5.81%)	1503 (7.69%)	1269 (5.33%)
BMI							
Underweight	514 (3.83%)	512 (4.43%)	855 (3.62%)	734 (4.32%)	632 (4.16%)	587 (3.95%)	770 (3.75%)
Normal weight	5619 (42.56%)	4692 (41.02%)	9153 (40.88%)	7675 (42.73%)	6782 (42.67%)	6576 (40.79%)	8959 (40.94%)
Overweight	3919 (28.21%)	3501 (29.49%)	7383 (31.62%)	6592 (33.31%)	6071 (33.82%)	6110 (34.51%)	8272 (35.33%)
Obese	1909 (13.01%)	2012 (15.30%)	3954 (15.35%)	4128 (19.64%)	3757 (19.36%)	3811 (20.75%)	4741 (19.99%)
N/A	1869 (12.40%)	1238 (9.76%)	1949 (8.53%)	-	-	-	-
Education							
Low	7498 (54.48%)	6547 (51.70%)	11,914 (49.22%)	9963 (49.19%)	8596 (45.85%)	7806 (43.09%)	9217 (39.49%)
Medium	4656 (34.61%)	4137 (37.17%)	8451 (38.50%)	6565 (37.02%)	6082 (38.52%)	6211 (39.36%)	8916 (40.78%)
High	1676 (10.90%)	1271 (11.13%)	2929 (12.28%)	2601 (13.79%)	2564 (15.62%)	3067 (17.55%)	4609 (19.73%)
Marital status							
Single/ divorced/ widowed	4159 (31.70%)	3789 (33.46%)	7644 (33.66%)	5968 (35.73%)	5330 (35.67%)	5358 (35.99%)	7693 (37.81%)
Married	9671 (68.30%)	8166 (66.54%)	15,650 (66.34%)	13,161 (64.27%)	11,912 (64.33%)	11,726 (64.01%)	15,049 (62.19%)
Lifestyle							
Rural	4217 (29.62%)	3185 (28.52%)	6058 (30.60%)	-	-	-	-
Urban	9613 (70.38%)	8770 (71.48%)	17,236 (69.40%)	-	-	-	-

**Table 2:** Lifestyle and social determinants of urinary incontinence.

Independent Variables	Odds Ratio [95% CI]	*p* Value *	Adjusted Odds Ratio ** [95% CI]	*p* Value *
Female				
Lifestyle				
Rural	Reference	Reference	Reference	Reference
Urban	0.61 [0.53–0.71]	**<0.001**	0.76 [0.66–0.88]	**<0.001**
Education				
Low	Reference	Reference	Reference	Reference
Medium	0.20 [0.16–0.26]	**<0.001**	0.57 [0.51–0.64]	**<0.001**
High	0.16 [0.06–0.41]	**<0.001**	0.38 [0.24–0.58]	**<0.001**
Marital status				
Single/divorced/widowed	Reference	Reference	Reference	Reference
Married	0.97 [0.91–1.04]	0.45	1.13 [1.05–1.22]	**0.001**
Depression symptom severity (PHQ-8)				
None	Reference	Reference	Reference	Reference
Mild	3.25 [2.98–3.55]	**<0.001**	2.10 [1.90–2.31]	**<0.001**
Moderate	5.99 [5.21–6.88]	**<0.001**	3.24 [2.68–3.92]	**<0.001**
Moderately severe	9.18 [7.66–11.00]	**<0.001**	4.61 [3.74–5.69]	**<0.001**
Severe	8.96 [7.19–11.16]	**<0.001**	3.26 [2.53–4.20]	**<0.001**
Use of depression medication	3.78 [2.76–5.19]	**<0.001**	1.27 [0.79–2.04]	0.32
Use of anxiety medication	2.45 [1.69–3.55]	**<0.001**	0.90 [0.56–1.44]	0.66
Male				
Lifestyle				
Rural	Reference	Reference	Reference	Reference
Urban	0.39 [0.31–0.47]	**<0.001**	0.59 [0.47–0.74]	**<0.001**
Education				
Low	Reference	Reference	Reference	Reference
Medium	0.17 [0.14–0.21]	**<0.001**	0.58 [0.50–0.67]	**<0.001**
High	0.21 [0.08–0.52]	**0.001**	0.47 [0.30–0.73]	**0.001**
Marital status				
Single/divorced/widowed	Reference	Reference	Reference	Reference
Married	2.47 [2.17–2.80]	**<0.001**	0.91 [0.79–1.05]	0.18
Depression symptom severity (PHQ-8)				
None	Reference	Reference	Reference	Reference
Mild	3.43 [2.93–4.02]	**<0.001**	2.19 [1.90–2.53]	**<0.001**
Moderate	6.70 [5.37–8.36]	**<0.001**	4.14 [3.11–5.51]	**<0.001**
Moderately severe	9.02 [6.66–12.22]	**<0.001**	4.48 [3.02–6.66]	**<0.001**
Severe	14.43 [10.50–19.84]	**<0.001**	6.11 [3.73–10.03]	**<0.001**
Use of depression medication	3.11 [1.81–5.33]	**<0.001**	1.20 [0.56–2.57]	0.63
Use of anxiety medication	4.14 [2.30–7.47]	**<0.001**	2.39 [1.20–4.79]	0.014

* After Bonferroni correction, a *p* value of less than 0.008 was considered significant. Significant values are highlighted in bold. ** Adjusted to age and the following comorbidities: depression, asthma, COPD, cardiac diseases, stroke, osteoarthritis, diabetes, and cirrhosis.

## Data Availability

The data used in this study were obtained from the Turkish Statistical Institute (TurkStat), but the accessibility of these data is limited due to certain constraints. The data were licensed specifically for this study and are not publicly available. However, authors can provide the data upon reasonable request and with included permission from TurkStat and the Regional Ethical Committee.

## Supplementary Materials

The following supporting information can be downloaded at: https://www.mdpi.com/article/10.3390/jcm14155213/s1.

## References

[1] Irwin D.E., Kopp Z.S., Agatep B., Milsom I., Abrams P. (2011). Worldwide Prevalence Estimates of Lower Urinary Tract Symptoms, Overactive Bladder, Urinary Incontinence and Bladder Outlet Obstruction. BJU Int..

[2] Buckley B.S., Lapitan M.C.M. (2010). Prevalence of Urinary Incontinence in Men, Women, and Children—Current Evidence: Findings of the Fourth International Consultation on Incontinence. Urology.

[3] Hannestad Y.S., Rortveit G., Sandvik H., Hunskaar S. (2000). A community-based epidemiological survey of female urinary incontinence: The Norwegian EPINCONT Study. J. Clin. Epidemiol..

[4] Thom D. (1998). Variation in Estimates of Urinary Incontinence Prevalence in the Community: Effects of Differences in Definition, Population Characteristics, and Study Type. J. Am. Geriatr. Soc..

[5] Markland A.D., Goode P.S., Redden D.T., Borrud L.G., Burgio K.L. (2010). Prevalence of Urinary Incontinence in Men: Results From the National Health and Nutrition Examination Survey. J. Urol..

[6] Subak L.L., Richter H.E., Hunskaar S. (2009). Obesity and Urinary Incontinence: Epidemiology and Clinical Research Update. J. Urol..

[7] Mishra G.D., Hardy R., Cardozo L., Kuh D. (2008). Body Weight through Adult Life and Risk of Urinary Incontinence in Middle-Aged Women: Results from a British Prospective Cohort. Int. J. Obes..

[8] Zhu L., Lang J., Liu C., Han S., Huang J., Li X. (2009). The Epidemiological Study of Women with Urinary Incontinence and Risk Factors for Stress Urinary Incontinence in China. Menopause.

[9] Hallock J.L., Handa V.L. (2016). The Epidemiology of Pelvic Floor Disorders and Childbirth. Obstet. Gynecol. Clin. N. Am..

[10] Weber M.A., Kleijn M.H., Langendam M., Limpens J., Heineman M.J., Roovers J.P. (2015). Local Oestrogen for Pelvic Floor Disorders: A Systematic Review. PLoS ONE.

[11] Ebbesen M.H., Hannestad Y.S., Midthjell K., Hunskaar S. (2007). Diabetes and Urinary Incontinence—Prevalence Data from Norway. Acta Obstet. Gynecol. Scand..

[12] Aoki Y., Brown H.W., Brubaker L., Cornu J.N., Daly J.O., Cartwright R. (2017). Urinary Incontinence in Women. Nat. Rev. Dis. Primers.

[13] Celik N., Celik S., Seyhan Z., Dasdelen M.F., Almas F., Albayrak S., Horuz R., Laguna P., de la Rosette J., Kocak M. (2024). The Relationship between Urinary Incontinence, Osteoarthritis, and Musculoskeletal System Disorders. J. Clin. Med..

[14] Fultz N.H., Herzog A.R. (2001). Self-Reported Social and Emotional Impact of Urinary Incontinence. J. Am. Geriatr. Soc..

[15] Hung K.J., Awtrey C.S., Tsai A.C. (2014). Urinary Incontinence, Depression, and Economic Outcomes in a Cohort of Women between the Ages of 54 and 65 Years. Obstet. Gynecol..

[16] Felde G., Engeland A., Hunskaar S. (2020). Urinary Incontinence Associated with Anxiety and Depression: The Impact of Psychotropic Drugs in a Cross-Sectional Study from the Norwegian HUNT Study. BMC Psychiatry.

[17] Reis A.M., Brito L.G.O., Lunardi A.L.B., Pinto E Silva M.P., Juliato C.R.T. (2021). Depression, Anxiety, and Stress in Women with Urinary Incontinence with or without Myofascial Dysfunction in the Pelvic Floor Muscles: A Cross-Sectional Study. Neurourol. Urodyn..

[18] Melville J.L., Delaney K., Newton K., Katon W. (2005). Incontinence Severity and Major Depression in Incontinent Women. Obstet. Gynecol..

[19] Nygaard I., Turvey C., Burns T.L., Crischilles E., Wallace R. (2003). Urinary Incontinence and Depression in Middle-Aged United States Women. Obstet. Gynecol..

[20] Coyne K.S., Sexton C.C., Irwin D.E., Kopp Z.S., Kelleher C.J., Milsom I. (2008). The Impact of Overactive Bladder, Incontinence and Other Lower Urinary Tract Symptoms on Quality of Life, Work Productivity, Sexuality and Emotional Well-being in Men and Women: Results from the EPIC Study. BJU Int..

[21] Glaser A.P., Mansfield S., Smith A.R., Helfand B.T., Lai H.H., Sarma A., Yang C.C., Taddeo M., Clemens J.Q., Cameron A.P. (2022). Impact of Sleep Disturbance, Physical Function, Depression and Anxiety on Male Lower Urinary Tract Symptoms: Results from the Symptoms of Lower Urinary Tract Dysfunction Research Network (LURN). J. Urol..

[22] Thom D.H., Haan M.N., Van Den Eeden S.K. (1997). Medically Recognized Urinary Incontinence and Risks of Hospitalization, Nursing Home Admission and Mortality. Age Ageing.

[23] Mishra G.D., Barker M.S., Herber-Gast G.-C., Hillard T. (2015). Depression and the Incidence of Urinary Incontinence Symptoms among Young Women: Results from a Prospective Cohort Study. Maturitas.

[24] Legendre G., Ringa V., Panjo H., Zins M., Fritel X. (2015). Incidence and Remission of Urinary Incontinence at Midlife: A Cohort Study. BJOG.

[25] Maserejian N.N., Minassian V.A., Chen S., Hall S.A., McKinlay J.B., Tennstedt S.L. (2014). Treatment Status and Risk Factors for Incidence and Persistence of Urinary Incontinence in Women. Int. Urogynecol. J..

[26] Perry S., McGrother C.W., Turner K., Leicestershire MRC Incontinence Study Group (2006). An Investigation of the Relationship between Anxiety and Depression and Urge Incontinence in Women: Development of a Psychological Model. Br. J. Health Psychol..

[27] Bogner H.R., O’Donnell A.J., de Vries H.F., Northington G.M., Joo J.H. (2011). The Temporal Relationship between Anxiety Disorders and Urinary Incontinence among Community-Dwelling Adults. J. Anxiety Disord..

[28] Felde G., Ebbesen M.H., Hunskaar S. (2017). Anxiety and Depression Associated with Urinary Incontinence. A 10-Year Follow-up Study from the Norwegian HUNT Study (EPINCONT). Neurourol. Urodyn..

[29] Melville J.L., Fan M.-Y., Rau H., Nygaard I.E., Katon W.J. (2009). Major Depression and Urinary Incontinence in Women: Temporal Associations in an Epidemiologic Sample. Am. J. Obstet. Gynecol..

[30] Dasdelen M.F., Almas F., Celik S., Celik N., Seyhan Z., Laguna P., Albayrak S., Horuz R., Kocak M., de la Rosette J. (2023). When Bladder and Brain Collide: Is There a Gender Difference in the Relationship between Urinary Incontinence, Chronic Depression, and Anxiety?. J. Clin. Med..

[31] Turkish Statistical Institute (2023). Türkiye Health Survey Micro Data Set 2022.

[32] Turkish Statistical Institute (2010). Health Survey 2008.

[33] UNESCO Institute for Statistics (2012). International Standard Classification of Education: ISCED 2011.

[34] Coyne K.S., Kvasz M., Ireland A.M., Milsom I., Kopp Z.S., Chapple C.R. (2012). Urinary Incontinence and Its Relationship to Mental Health and Health-Related Quality of Life in Men and Women in Sweden, the United Kingdom, and the United States. Eur. Urol..

[35] Gacci M., Sakalis V.I., Karavitakis M., Cornu J.-N., Gratzke C., Herrmann T.R.W., Kyriazis I., Malde S., Mamoulakis C., Rieken M. (2022). European Association of Urology Guidelines on Male Urinary Incontinence. Eur. Urol..

[36] Chen X., Jiang S., Yao Y. (2023). Association between Obesity and Urinary Incontinence in Older Adults from Multiple Nationwide Longitudinal Cohorts. Commun. Med..

[37] Shang X., Fu Y., Jin X., Wang C., Wang P., Guo P., Wang Y., Yan S. (2023). Association of Overweight, Obesity and Risk of Urinary Incontinence in Middle-Aged and Older Women: A Meta Epidemiology Study. Front. Endocrinol..

[38] Joinson C., Drake M.J., Fraser A., Tilling K., Heron J. (2025). Bidirectional Relationships between Depression, Anxiety and Urinary Symptoms in Women: A Prospective Cohort Study. J. Affect. Disord..

[39] Thoits P., Hannan M. (1979). Income and Psychological Distress: The Impact of an Income-Maintenance Experiment. J. Health Soc. Behav..

[40] Diener E., Sandvik E., Seidlitz L., Diener M. (1993). The Relationship between Income and Subjective Well-Being: Relative or Absolute?. Soc. Indic. Res..

[41] Sareen J., Afifi T.O., McMillan K.A., Asmundson G.J.G. (2011). Relationship Between Household Income and Mental Disorders. Arch. Gen. Psychiatry.

[42] Lund C., Cois A. (2018). Simultaneous Social Causation and Social Drift: Longitudinal Analysis of Depression and Poverty in South Africa. J. Affect. Disord..

[43] Li C., Ning G., Wang L., Chen F. (2022). More Income, Less Depression? Revisiting the Nonlinear and Heterogeneous Relationship between Income and Mental Health. Front. Psychol..

[44] Lin Y., Lin Y., Wu I., Chang Y. (2021). Urinary Incontinence and Its Association with Socioeconomic Status among Middle-aged and Older Persons in Taiwan: A Population-based Study. Geriatr. Gerontol. Int..

[45] Lee J.A., Johns T.S., Melamed M.L., Tellechea L., Laudano M., Stern J.M., Abraham N.E. (2020). Associations between Socioeconomic Status and Urge Urinary Incontinence: An Analysis of NHANES 2005 to 2016. J. Urol..

[46] Viseu J., Leal R., de Jesus S.N., Pinto P., Pechorro P., Greenglass E. (2018). Relationship between Economic Stress Factors and Stress, Anxiety, and Depression: Moderating Role of Social Support. Psychiatry Res..

[47] Ni M.Y., Yao X.I., Leung K.S.M., Yau C., Leung C.M.C., Lun P., Flores F.P., Chang W.C., Cowling B.J., Leung G.M. (2020). Depression and Post-Traumatic Stress during Major Social Unrest in Hong Kong: A 10-Year Prospective Cohort Study. Lancet.

[48] Duarte F., Jiménez-Molina Á. (2024). Exploring the Impact of Social Protest on Mental Health: A Study of the 2019 “Social Uprising” in Chile. Soc. Sci. Med..

[49] Rai D., Zitko P., Jones K., Lynch J., Araya R. (2013). Country-and Individual-Level Socioeconomic Determinants of Depression: Multilevel Cross-National Comparison. Br. J. Psychiatry.

[50] Cifuentes M., Sembajwe G., Tak S., Gore R., Kriebel D., Punnett L. (2008). The Association of Major Depressive Episodes with Income Inequality and the Human Development Index. Soc. Sci. Med..

[51] Rortveit G., Daltveit A.K., Hannestad Y.S., Hunskaar S. (2003). Urinary Incontinence after Vaginal Delivery or Cesarean Section. N. Engl. J. Med..

[52] (2023). Turkish Statistical Institute Birth Statistics. https://data.tuik.gov.tr/Bulten/Index?p=Birth-Statistics-2023-53708&dil=2.

[53] Birinci Ş., Parpucu Ü.M. (2023). When a Caesarean Section Is Necessary: Analysis of Cesarean Sections Performed in the Republic of Turkey in 2022 in Accordance with the World Health Organization Multi-Country Research Guidelines. J. Turk. Soc. Obstet. Gynecol..

[54] Global Burden of Disease Collaborative Network Global Burden of Disease Study 2021 (GBD 2021) Results. https://vizhub.healthdata.org/gbd-compare/.

[55] Ülgü M.M., Gülkesen K.H., Akünal A., Ayvali M.O., Zayim N., Birinci Ş., Balci M.K. (2023). Characteristics of Diabetes Mellitus Patients in Turkey: An Analysis of National Electronic Health Records. Turk. J. Med. Sci..

