# Supplementary material for: Exploring the Association Between Urinary Incontinence and Depression Based on a Series of Large-Scale National Health Studies in Türkiye
Source: J Clin Med. 2025 Jul 23;14(15):5213. doi: 10.3390/jcm14155213 (PMC12347464; doi:10.3390/jcm14155213)
Supplement: Supplementary file 1 [file jcm-14-05213-s001.zip › supplement Figure S1.pdf]

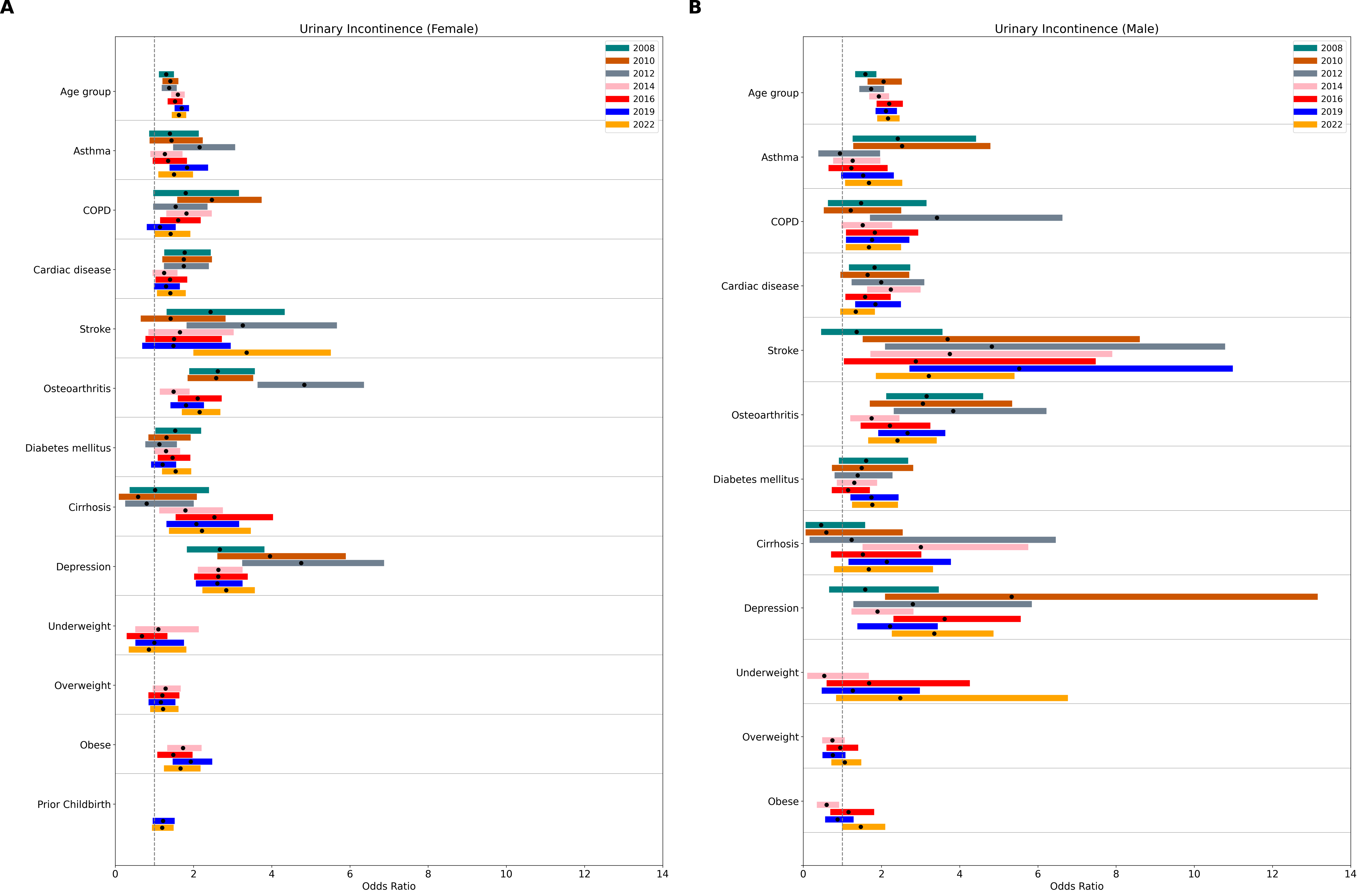

Supplementary Figure S1: Detailed UI odds ratios by year. Separate multivariable logistic regressionmodels were fitted for each year across both sexes. Different colors represent different years, as specifiedin the legend. Black dots indicate the odds ratios, and colored bars show the 95% confidence intervals.
