# Supplementary material for: Exploring the Association Between Urinary Incontinence and Depression Based on a Series of Large-Scale National Health Studies in Türkiye
Source: J Clin Med. 2025 Jul 23;14(15):5213. doi: 10.3390/jcm14155213 (PMC12347464; doi:10.3390/jcm14155213)
Supplement: Supplementary file 1 [file jcm-14-05213-s001.zip › Supplementary_Tables.pdf]

Supplementary Table S1. Age-standardized prevalence of chronic diseases in both sexes.

Disease	**2008 (%)**	**2010 (%)**	**2012 (%)**	**2014 (%)**	**2016 (%)**	**2019 (%)**	**2022 (%)**
				**Female**			
**Asthma**	7.6	7.99	8.2	12.73	12.5	14.16	12.46
**Cirrhosis**	1.38	0.78	0.66	2.52	2.36	2.1	1.9
**COPD**	3.02	5.33	4.03	11.37	10.93	11.06	9.43
**Coronary heart disease**	9.82	6.5	6.55	12.44	9.61	9.74	9.0
**Depression**	6.03	4.44	3.29	15.13	10.12	13.01	9.85
**Diabetes**	10.79	11.4	11.84	15.3	14.95	15.58	17.34
**Hypertension**	29.22	26.21	26.82	29.69	28.67	28.07	26.89
**MI**	2.82	1.23	1.09	2.89	2.89	2.77	3.14
**Osteoarthritis**	24.27	17.19	13.6	15.35	15.11	18.08	13.34
**Stroke**	1.86	1.8	1.6	1.23	1.14	1.11	1.13
**UI**	8.57	6.7	4.76	14.25	10.96	13.97	9.56
				**Male**			
**Asthma**	4.42	5.23	5.0	6.82	6.95	7.37	6.47
**Cirrhosis**	1.46	0.7	0.57	1.84	1.44	1.92	1.34
**COPD**	2.95	5.83	3.75	8.12	7.67	6.95	6.64
**Coronary heart disease**	8.09	7.4	6.52	10.28	8.52	8.99	8.16
**Depression**	2.1	1.53	1.13	7.86	5.19	6.03	4.8
**Diabetes**	7.5	7.55	8.56	10.12	10.29	10.99	12.15
**Hypertension**	16.48	16.34	14.65	18.06	17.31	17.91	17.17
**MI**	3.77	2.99	2.0	3.42	3.5	3.72	3.74
**Osteoarthritis**	11.97	8.07	5.97	8.7	7.87	9.83	6.14
**Stroke**	1.68	2.09	1.67	1.49	1.45	1.11	1.52
**UI**	5.1	3.8	2.83	8.78	7.56	9.11	6.28

Supplementary Table S2. Adjustment for previous pregnancy in the multivariable model attenuated the effect of marital status (In comparison to Table 2 in main text).

Year	Variable	OR	CI-Low	CI-High	P value
2019	Age group	1.71	1.61	1.81	<0.01
	Depression	2.62	2.15	3.19	<0.01
	Asthma	1.89	1.50	2.37	<0.01
	COPD	1.17	0.91	1.51	0.22
	Cardiac_disease	1.38	1.14	1.68	0.00
	Stroke	1.36	0.71	2.62	0.36
	Osteoarthritis	1.85	1.52	2.24	<0.01
	Diabetes	1.32	1.08	1.61	0.01
	Cirrhosis	2.07	1.40	3.06	<0.01
	**Married**	**0.98**	**0.82**	**1.17**	**0.83**
	**Pregnancy**	**1.29**	**1.09**	**1.52**	**<0.01**
2022	Age group	1.65	1.55	1.75	<0.01
	Depression	2.84	2.31	3.50	<0.01
	Asthma	1.53	1.20	1.96	<0.01
	COPD	1.42	1.09	1.86	0.01
	Cardiac_disease	1.49	1.21	1.82	<0.01
	Stroke	3.37	2.09	5.44	<0.01
	Osteoarthritis	2.20	1.81	2.67	<0.01
	Diabetes	1.62	1.34	1.96	<0.01
	Cirrhosis	2.23	1.46	3.40	<0.01
	**Married**	**1.02**	**0.86**	**1.22**	**0.78**
	**Pregnancy**	**1.24**	**1.05**	**1.46**	**0.01**

Supplementary Table S3. UI prevalence in different income levels

Income Level	**2008 N (%)**	**2010 N (%)**	**2012 N (%)**	**2014 N (%)**	**2016 N (%)**	**2019 N (%)**	**2022 N (%)**
**1**	71 (7.25%)	55 (5.13%)	32 (4.8%)	754 (11.44%)	434 (9.36%)	43 (15.54%)	18 (12.09%)
**2**	68 (8.04%)	60 (4.89%)	15 (2.5%)	303 (6.61%)	347 (5.9%)	21 (10.98%)	10 (10.81%)
**3**	25 (3.55%)	46 (3.55%)	12 (2.28%)	225 (6.49%)	191 (5.43%)	22 (16.67%)	3 (4.53%)
**4**	25 (5.13%)	71 (4.39%)	16 (2.1%)	206 (5.63%)	135 (3.87%)	20 (20.51%)	9 (18.72%)
**5**	29 (6.51%)	38 (2.44%)	30 (2.79%)	131 (4.03%)	112 (3.94%)	15 (21.68%)	21 (19.27%)
**6**	20 (4.38%)	56 (3.35%)	12 (1.14%)			10 (4.61%)	4 (11.45%)
**7**	8 (2.17%)	17 (1.71%)	20 (1.9%)			18 (10.07%)	14 (16.66%)
**8**	21 (5.4%)	45 (2.69%)	27 (1.76%)			8 (9.17%)	17 (13.77%)
**9**	5 (2.94%)	24 (2.3%)	22 (1.89%)			8 (7.54%)	8 (6.77%)
**10**	12 (4.35%)	33 (2.17%)	26 (1.79%)			5 (6.95%)	4 (3.33%)
**11**						10 (6.42%)	14 (7.82%)
**12**						4 (4.25%)	13 (5.05%)
**13**						5 (4.92%)	11 (5.67%)
**14**						12 (9.75%)	1 (0.88%)
**15**						14 (12.9%)	13 (10.27%)
**16**						8 (5.16%)	13 (7.16%)
**17**						7 (10.85%)	6 (2.9%)
**18**						6 (6.98%)	15 (4.22%)
**19**						3 (2.33%)	7 (3.3%)
**20**						3 (7.01%)	13 (2.77%)
**X**	38.35	58.59	38.75	234.67	197.92	73.50	112.59
**P value***	<0.001	<0.001	<0.001	<0.001	<0.001	<0.001	<0.001

* After Bonferroni correction, P value less than 0.007 was considered significant

Supplementary Table S4. Classification of income levels by years

Income Level	2008 N (%)	2010 N (%)	2012 N (%)	2014 N (%)	2016 N (%)	2019 N (%)	2022 N (%)
**1**	<350	<350	<350	<1080	<1264	<992	<1646
**2**	351-500	351-500	351-500	1081-1550	1265-1814	993 - 1298	1647 - 2212
**3**	501-620	501-620	501620	1551-2710	1815-2540	1299 - 1500	2213 - 2570
**4**	621-750	621-750	621-750	2711-3180	2541-3721	1501 - 1668	2571 - 2883
**5**	751-900	751-900	751-900	>3181	>3722	1669 - 1849	2884 - 3172
**6**	901-1100	901-1100	901-1100			1850 - 2025	3173 - 3472
**7**	1101-1300	1101-1300	1101-1300			2026 - 2214	3473 - 3774
**8**	1301-1700	1301-1700	1301-1700			2215 - 2424	3775 - 4111
**9**	1701-2300	1701-2300	1701-2300			2425 - 2656	4112 - 4462
**10**	>2301	>2301	>2301			2657 - 2892	4463 - 4852
**11**						2893 - 3144	4853 - 5268
**12**						3145 - 3398	5269 - 5713
**13**						3399 - 3695	5714 - 6199
**14**						3696 - 4052	6200 - 6794
**15**						4053 - 4492	6795 - 7510
**16**						4493 - 5052	7511 - 8372
**17**						5053 - 5784	8373 - 9539
**18**						5785 - 6890	9540 - 11356
**19**						6891 - 8912	11357 - 14812
**20**						>8913	>14813

Supplementary Table S5. Model summaries of Interrupted Time Series analysis

	Estimate Std.	Error	t value	Pr(>|t|)
Depression				
Intercept	4.1987	1.1230	3.739	0.03337
time	-0.4952	0.5245	-0.944	0.41481
event	8.1685	1.0682	7.647	0.00464
time_event	-0.2575	0.5374	-0.479	0.66464
UI				
Intercept	7.9541	0.5041	15.780	0.00399
time	-1.2777	0.2360	-5.414	0.03246
event	7.0269	0.4953	14.188	0.00493
time_event	0.9364	0.2447	3.827	0.06201
